# Phenolic Content, Amino Acids, Volatile Compounds, Antioxidant Capacity, and Their Relationship in Wild Garlic (*A. ursinum* L.)

**DOI:** 10.3390/foods12112110

**Published:** 2023-05-24

**Authors:** Tvrtko Karlo Kovačević, Nikola Major, Marta Sivec, Dijana Horvat, Marina Krpan, Mirjana Hruškar, Dean Ban, Nina Išić, Smiljana Goreta Ban

**Affiliations:** 1 Department of Agriculture and Nutrition, Institute of Agriculture and Tourism, K. Hugues 8, 52440 Poreč, Croatiasmilja@iptpo.hr (S.G.B.); 2 Centre of Excellence for Biodiversity and Molecular Plant Breeding, Svetošimunska 1, 10000 Zagreb, Croatia; 3 Križevci College of Agriculture, Milislava Demerca 4, 48260 Križevci, Croatia; 4 Department of Food Quality Control, Faculty of Food Technology and Biotechnology, University of Zagreb, Pierottijeva 6, 10000 Zagreb, Croatia

**Keywords:** bear’s garlic, ramson, buckrams, plant organ, HS-GC/MS, biodiversity, bioactive compounds

## Abstract

*Allium ursinum* L. is a wild relative of garlic, and it is abundant in many antioxidant compounds. Sulfur compounds, primarily cysteine sulfoxides (CSOs), are converted through several reactions into various volatile molecules, which are considered the principal flavor compounds of Alliums. In addition to secondary metabolites, wild garlic is abundant in primary compounds, such as amino acids, which serve not only as building blocks for the health-promoting sulfur compounds but also as antioxidants. The aim of this study was to investigate the link between individual amino acid contents, the total phenolic content, and the profile of volatile compounds as well as their influence on the antioxidant capacity of both the leaves and bulbs of wild garlic populations in Croatia. Both univariate and multivariate methods were used to study the differences in the phytochemical compositions among the wild garlic plant organs and the link between individual compounds and antioxidant capacity. Both the plant organ and location, as well as their interaction, have a significant impact on the content of total phenolic content, amino acids, volatile organic compounds, and the antioxidant capacity of wild garlic.

## 1. Introduction

*Allium ursinum* L. is a plant whose species name is of Latin origin, being derived from “*ursus*”, which means bear. Often, it is related to folk tales, according to which bears consume this plant to regain their strength after awakening from winter hibernation by removing toxins from the body [1]. *Allium* species have been used in traditional medicine for many centuries due to their widespread distribution and popularity as edible and medicinal plants [2]. In the past few years, interest in *Allium ursinum* L. has significantly grown [2]. Since wild garlic is a relative of garlic, they share pharmacological properties due to their similar phytochemical compositions [3,4].

The aroma and pharmacological properties of wild garlic and other *Allium* plants are primarily attributed to the phytochemicals containing sulfur atoms. Such sulfur-containing compounds are known as organosulfur compounds (OSC) [5]. Furthermore, sulfoxides are described as non-volatile sulfur secondary metabolites, which are found compartmentalized in the cytoplasm, in intact cells of the plant. Disruption of the cellular structure by mechanical force and/or by heat results in tissue damage, which leads to a release of the hydrolytic enzyme alliinase, found in the vacuole, and sulfoxides from their compartmentalized sections, resulting in subsequent hydrolysis, which gives rise to many volatile compounds, such as thiosulfinates and sulfides, which are the principal flavor compounds of *Allium* plants [6].

Amino acids are the main precursors for the formation of cysteine sulfoxides and glutamyl peptides, and they also function as building blocks for proteins and enzymes as well [4]. Moreover, not only can S-amino acids be found in wild garlic extracts, but various ubiquitous amino acids have been reported by conducted studies, such as arginine, glutamic acid, aspartic acid, methionine, threonine, valine, leucine, and proline [7]. The role of amino acids is not only as building blocks for large molecules, but they also act as antioxidants, especially sulfur-containing amino acids, such as methionine [8].

In addition to flavor-carrying organosulfur compounds and amino acids, other compounds, such as polyphenols, greatly contribute to the antioxidant properties of wild garlic [5,9,10]. Polyphenols are a group of bioactive compounds commonly found in vegetables, fruits, and many other food sources [11].

Wild edible plants, and wild garlic as well, are plants that have not been cultivated or domesticated but are available from their natural habitat and are used as food or medicinal herbs [12]. If not used for nutrition, *A. ursinum* has traditionally been used for medical purposes, equally valuable as common garlic. It has often been used for removing toxins from the body and in preventing cardiovascular diseases [13,14]. Benkeblia et al. [15] and Pavlović et al. [16] have reported high antioxidant and radical scavenging activity in *A. ursinum* extracts.

All bioactive compounds are subject to change depending on the growing environment, such as temperature, humidity, and even altitude, but also on the physiological maturity of the plant [17]. The reallocation of bioactive compounds also differs among the plant organs. *Allium* species are well-adapted to various climatic conditions, but the majority of the species prefers arid and moderately humid climates and open, sunny areas [17]. On the other hand, wild garlic mostly grows in the forests of Europe and Asia, preferably next to water streams [1]. It prefers humid soils and humus and areas with full or semi-shade [4,9]. In this regard, wild garlic is an important research topic, especially in light of global climate change and the preservation of biodiversity [18].

The Mediterranean Basin is considered an important area of biodiversity since it is host to many wild species, and access to such biological resources is gaining increasing significance [7].

The aim of this study was to investigate the links among individual amino acid contents, the total phenolic content, and the profile of volatile compounds as well as their influence on the antioxidant capacity of both leaves and bulbs of the wild garlic populations in Croatia. The present study also investigated the biodiversity of wild garlic plants originating from eight locations across Croatia to evaluate how different environments can affect both the leaf and bulb phytochemical contents and the antioxidant capacity.

## 2. Materials and Methods

### 2.1. Reagents

The reagents used in this study were as follows: HPLC-grade methanol (J.T. Baker), ethanol (J.T. Baker), HPLC-grade acetonitrile (VWR, Darmstadt, Germany), HPLC-grade water (VWR, Darmstadt, Germany) Folin–Ciocalteu reagent (Sigma-Aldrich, Saint-Quentin-Fallavier, France), 6% (*w*/*v*) anhydrous sodium carbonate (Gram-Mol, Zagreb, Croatia), 2,2-diphenyl-1-picrylhydrazyl (DPPH) (Sigma-Aldrich, Saint-Quentin-Fallavier, France), sodium acetate trihydrate (Lach-Ner, Neratovice, Czech Republic), 2,4,6-tris(2-pyridyl)-s-triazine (TPTZ) (Alfa Aesar, Ward Hill, MA, USA), glacial acetic acid (Macron, Crespellano, Italy), concentrated hydrochloric acid (Carlo Erba, Milano, Italy), iron (III) chloride hexahydrate (Kemika, Zagreb, Croatia), fluorescein disodium salt (Alfa Aesar, Ward Hill, MA, USA), 2,2′-azobis(2-methylpropionamidine) dihydrochloride (AAPH) (Acros Organics, Geel, Belgium), 10% (*w*/*v*) sodium chloride (VWR, Darmstadt, Germany), potassium dihydrogen phosphate (Gram-Mol, Zagreb, Croatia), dipotassium hydrogen phosphate (Gram-Mol, Zagreb, Croatia), o-phthalaldehyde (OPA), methiopropamine (MPA), 9-fluorenylmethyl chloroformate (FMOC). The chemicals used as standards were: gallic acid (Acros Organics, Geel, Belgium), 6-hydroxy-2,5,7,8-tetramethylchroman-2-carboxylic acid (Sigma-Aldrich, Saint-Quentin-Fallavier, France), amino acid standard (Sigma-Aldrich, Saint-Quentin-Fallavier, France), norvaline (Acros Organics), sarcosine (Alfa Aesar, Ward Hill, MA, USA), 2-octanol as an internal standard for gas chromatography, and diallyl sulfide/disulfide/trisulfide (Cayman Chemical Company, Ann Arbor, MI, USA). 

### 2.2. Plant Material

The eight *A. ursinum* populations used in this study were collected in situ from different habitats across the Republic of Croatia during the spring of 2021 (Supplementary Table S1). The plants were harvested in the vegetative stage before flower stalk development. The bulbs and leaves of the fresh plant samples were separated (three biological replicates per bulb/leaf), and after harvesting, they were flash-frozen in liquid nitrogen and kept at −80 °C until further processing. The frozen bulbs and leaves were placed in a freeze dryer (Labogene ScanVac CoolSafe, Allerød, Denmark) and lyophilized over a period of 48 h. The lyophilized samples were ground to powder (0.2 mm) using an ultra-centrifugal mill (Retsch ZM200, Haan, Germany). The dried plant material (75 mg) was extracted with 1.5 mL of 80:20 methanol/water (*v*/*v*) at 30 °C over a period of 30 min in a 600 W ultrasonic bath (MRC 250H, Holon, Israel). The samples were centrifuged at 16,000× *g* for 10 min (Domel Centric 350, Železniki, Slovenia), and the supernatant was filtered through a 0.22 µm nylon filter. The samples were stored at −80 °C until further analysis.

### 2.3. Total Phenolic Content and Total Antioxidant Capacity

A total phenolic content assay was performed according to Singleton and Rossi [19], with slight modifications. The methanolic extracts (100 µL) were mixed with 100 µL of freshly prepared 0.2 M Folin–Ciocalteu reagent and with 100 µL of 6% solution of sodium carbonate, which was added 1 min after adding the Folin–Ciocalteu reagent. The absorbance was read at 750 nm (Tecan Infinite 200 Pro M Nano+, Männedorf, Switzerland) after 60 min of reaction time at 25 °C. The results were calculated against a standard curve of gallic acid (y = 4.6244x − 0.0798; serial dilutions of gallic acid—20, 40, 60, 80, 100 mg/L; coefficient of determination, R^2^ = 0.9998). The results are expressed as mg GAE/g DW.

The total antioxidant capacity was evaluated using the ferric-reducing antioxidant power (FRAP) assay, the DPPH radical scavenging activity assay, and the oxygen radical absorbance capacity (ORAC) assay.

The FRAP assay was performed according to Benzie and Strain [20], with some modifications. Briefly, 100 µL of the methanolic extract was mixed with 200 µL of freshly prepared FRAP reagent, and after 10 min of reaction time at 25 °C, the antioxidant capacity was measured by reading the absorbance at 593 nm (Tecan Infinite 200 Pro M Nano+, Männedorf, Switzerland). The FRAP values were calculated against a Trolox calibration curve (y = 7.8989x − 0.0179; serial dilutions of Trolox—20, 40, 60, 80, 100 µM; coefficient of determination, R^2^ = 0.9999), and all values are expressed as µmol TE/g DW. 

The DPPH radical scavenging activity assay was performed according to Brand-Wiliams et al. [21], with slight modifications. Briefly, 100 µL of the methanolic extract sample was mixed with 200 µL of freshly prepared 0.02 M DPPH radical. After 30 min of reaction time at 25 °C, the DPPH radical scavenging ability was evaluated by reading the absorbance at 517 nm (Tecan Infinite 200 Pro M Nano+, Männedorf, Switzerland). The DPPH radical scavenging ability values were calculated against a standard curve of Trolox (y = −13.003x + 13.066; serial dilutions of Trolox—20, 40, 60, 80, 100 µM; coefficient of determination, R^2^ = 0.9998). The values are expressed as µmol TE/g DW. 

The ORAC assay was performed according to Ou et al. [22], with some modifications. Briefly, the methanol extracts (37.5 µL) were mixed with 225 µL of freshly prepared 4 µM fluorescein solution and incubated for 30 min at 37 °C. Freshly made AAPH (37.5 µL) was added to the mixture, and the reaction was monitored over 120 min with the excitation and emission wavelengths of 485 nm and 528 nm, respectively (Tecan Infinite 200 Pro M Nano+, Männedorf, Switzerland). The results were calculated against a standard curve of Trolox (y = 0.0469x − 0.0045; serial dilutions of Trolox—4, 8, 12, 16, 20 µM; coefficient of determination, R^2^ = 0.9997). The values are expressed as nmol TE/g DW.

### 2.4. Amino Acids

Amino acid analysis was performed according to Buha et al. [23], with slight modifications. A volume of 100 µL of supernatant was taken and transferred into a clean HPLC vial, to which 5 µL of internal standard (10 µM) was added. The analysis was performed on a UPLC instrument, which consisted of a solvent delivery unit (Nexera LC-40DX3, Shimadzu, Kyoto, Japan), an autosampler (Nexera SIL-40CX3, Shimadzu, Kyoto, Japan), an oven (Nexera CTO-40C, Shimadzu, Kyoto, Japan), and a fluorescence detector (RF-20AXS, Shimadzu, Kyoto, Japan). An automated pre-column derivatization process was conducted using OPA and MPA for the primary and FMOC for the secondary amino acids. Amino acid separation was performed by injecting 5 µL of the sample extract spiked with internal standards (norvaline and sarcosine for the primary and secondary amino acids, respectively) on a 250 mm × 4 mm, C18, 4 µm particle-size column (Agilent, Santa Clara, CA, USA), with the mobile phase flow set to 0.8 mL/min, and with gradient elution with borate buffer (pH 8.2) as mobile phase A and methanol/acetonitrile/water (45:45:10, *v*/*v*/*v*) as mobile phase B. The gradients of the mobile phases were as follows: from 0 to 40 min, 57% B + 43% A; from 40 to 44 min, 100% B; from 44 to 54 min, 100% B; from 54 to 55 min, 2% B + 98% A; from 55 to 60 min, 2% B + 98% A. The primary amino acids were quantified at the excitation and emission wavelengths of 340 nm and 450 nm, respectively, while the secondary amino acids were quantified at the excitation and emission wavelengths of 260 nm and 305 nm, respectively. The total analysis time was 60 min. The identification and quantification of the amino acids were performed against external standards.

### 2.5. Volatile Organic Compounds

An assay was performed according to Molina-Calle et al. [24], with some modifications. Previously ground and dry-frozen samples of bulbs and leaves were weighed (250 mg) and transferred to twenty-milliliter headspace (HS) vials. A volume of 7950 µL of 10% (*w*/*v*) sodium chloride was added, along with 50 µL of internal standard (2-octanol). Before a 1 min vortex, the vials were sealed with 20 mm magnetic vial caps (LLG Labware, Meckenheim, Germany) and 20 mm transparent blue silicone/PTFE septa (LLG Labware, Meckenheim, Germany). The analysis was performed on a GCMS instrument, which consisted of an autosampler (AOC-6000, Shimadzu, Kyoto, Japan), a gas chromatograph (Nexis GC-2030, Shimadzu, Kyoto, Japan) coupled to a triple quadrupole mass detector (GCMS-TQ8040 NX, Shimadzu, Kyoto, Japan). The sample was incubated for 45 min at 60 °C with agitation, after which separation was performed on a Rxi-5Sil MS column (Restek, Bellefonte, PA, USA) by a splitless injection of 2 mL of the headspace content directly into the column, with a helium flow set to 1 mL/min. The oven temperature was programmed as follows: initial temperature of 40 °C (held for 5 min), increased to 130 °C, 3 °C/min (held for 5 min); increased to 220 °C, 4 °C/min (held for 5 min); increased to 260 °C, 15 °C/min; 260 °C (held for 3 min). The total analysis time was 68 min. The triple quadrupole mass spectrometer was operated in the Q3 scan mode, for which the instrumental parameters were set as follows: ion source temperature of 280 °C, interface temperature of 300 °C, electron energy of −70 eV, with the data acquisition rate set to an *m*/*z* ratio between 40 and 350. The obtained areas under the peak were normalized against the internal standard. Compounds were identified using the NIST17 database and retention indices. For each compound detected, its concentration was calculated against a standard (diallyl sulfide, diallyl disulfide, or diallyl trisulfide).

### 2.6. Statistical Analysis

The obtained data were further processed with a factorial analysis of variance (ANOVA). Differences among the means were compared using Fischer’s LSD post-hoc test at *p* ≤ 0.05. The obtained results for the volatile profile and amino acids of wild garlic were analyzed by the MetaboAnalyst R package [25]. In addition, a multivariate statistical analysis, partial least squares—discriminant analysis (PLS-DA), was employed for determining the discrimination between bulb and leaf samples. Pearson’s correlations between amino acids, VOCs, TPC, and AOC were investigated as well. Unless otherwise stated, all statistical analyses were performed using Statistica 13.4 (TIBCO Inc., Palo Alto, CA, USA).

## 3. Results

### 3.1. Relationships among Total Phenolic Content, Amino Acids, Volatile Compounds, and Antioxidant Capacity in Both Leaves and Bulbs of Wild Garlic

**Figure 1:**
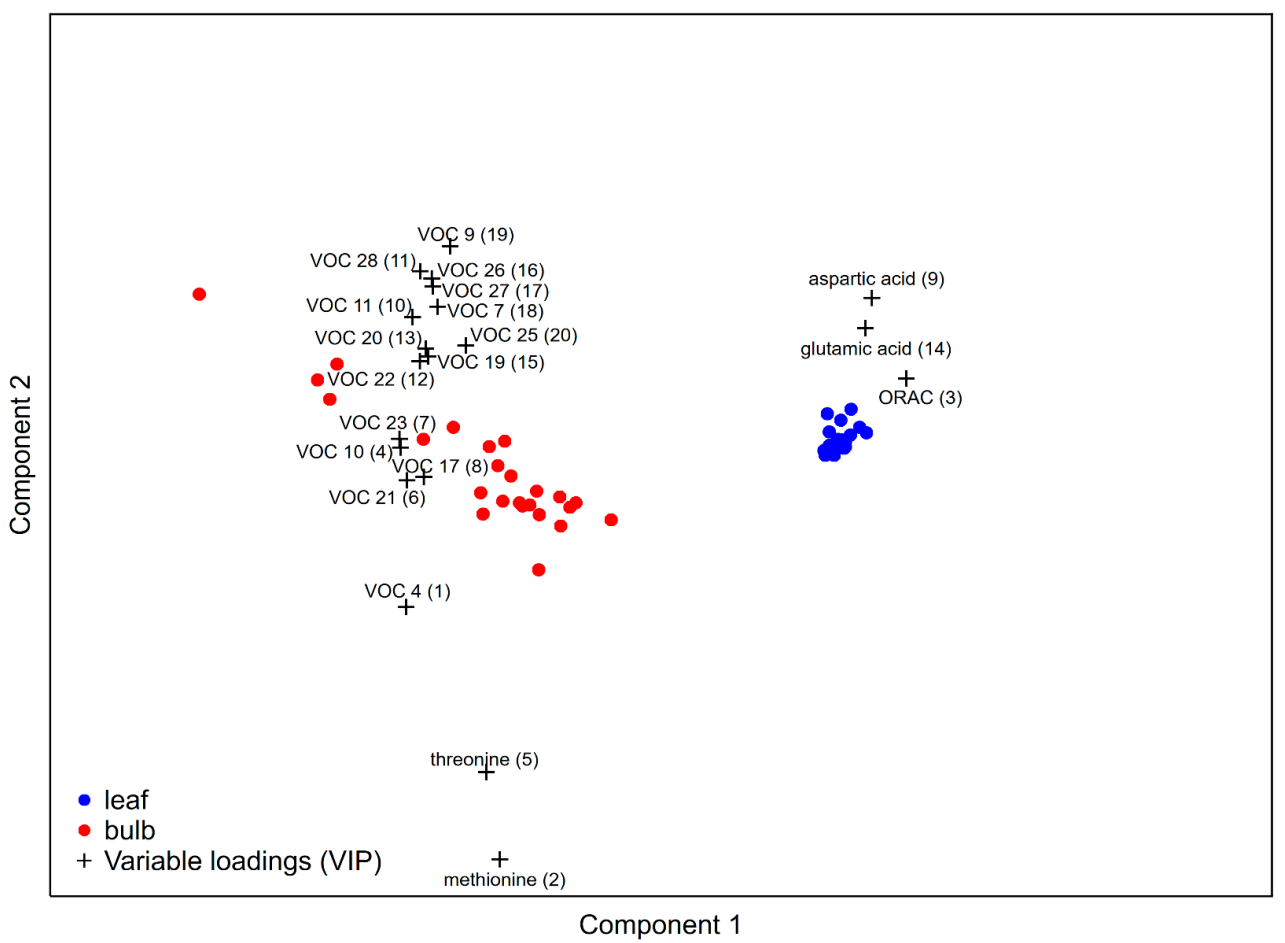
PLS-DA comparison of the investigated wild garlic bulb and leaf samples. (VOC 4: allyl propyl sulfide; VOC 7: methyl-2-propenyl disulfide; VOC 9: (*E*)-methyl-1-propenyl disulfide; VOC 10: 1,2-dithiole; VOC 11: diallyl disulfide; VOC 17: methyl-2-propenyl trisulfide; VOC 19: (*Z*)-methyl-1-propenyl trisulfide; VOC 20: (*E*)-methyl-1-propenyl trisulfide; VOC 21: 3-vinyl-1,2-dithi-4-ene; VOC 22: 4H-1,2,3-trithiin; VOC 23: dimethyl tetrasulfide; VOC 25: allyl propyl trisulfide; VOC 26: allyl (*Z*)-prop-1-enyl trisulfide; VOC 27: allyl (*E*)-prop-1-enyl trisulfide; VOC 28: methyl-1,2,3,4-tetrathiane). The number in parenthesis indicates the Variable in Projection (VIP) ranking in descending order (from most important ranked 1 to least important ranked 20).

Partial least squares—discriminant analysis was used to determine the differential allocation of phytochemical compounds between the leaves and bulbs of wild garlic. As seen in Figure 1, the obtained model showed the phytochemical compounds that were differently distributed between the bulbs and leaves. The variable importance in the projection (VIP) scores shows that the phytochemical markers that differed the most, in descending order, were allyl propyl sulfide (VOC 4), methionine, ORAC, 1,2-dithiole (VOC 10), threonine, 3-vinyl-1,2-dithi-4-ene (VOC 21), dimethyl tetrasulfide (VOC 23), methyl-2-propenyl trisulfide (VOC 17), aspartic acid, diallyl disulfide (VOC 11), methyl-1,2,3,4-tetrathiane (VOC 28), 4H-1,2,3,-trithiin (VOC 22), (*E*)-methyl-1-propenyl trisulfide (VOC 20), glutamic acid, (*Z*)-1-methyl-1-propenyl trisulfide (VOC 19), allyl (*Z*)-prop-1-enyl trisulfide (VOC 26), allyl (*E*)-prop-1-enyl trisulfide (VOC 27), methyl-2-propenyl disulfide (VOC 7), (*E*)-methyl-1-propenyl disulfide (VOC 9), and allyl propyl trisulfide (VOC 25). The reallocation of volatile compounds in the bulb compared to the leaf was more pronounced for diallyl- disulfide, trisulfide, and tetrasulfide isomers and several compounds with cyclic aromatic structures with sulfur atoms. For the amino acids, threonine and the sulfur-containing methionine were mostly allocated to the bulb, while glutamic and aspartic acids were more characteristic for the leaves of wild garlic. The leaf samples of the investigated wild garlic populations were characterized by higher ORAC values compared to the bulb samples.

The TPC showed a significant positive correlation with several amino acids, namely threonine, methionine, phenylalanine, isoleucine, leucine, and lysine, and a significant negative correlation with glutamic acid (Table 1). All investigated amino acids were significantly correlated to volatile organic compounds (Table 1). Aspartic acid, glutamic acid, histidine, glycine, and cystine correlated negatively, while threonine, arginine, methionine, and isoleucine correlated positively with volatile compounds. Tyrosine and valine correlated negatively with all but dimethyl trisulfide and dially trisulfide, respectively, which correlated positively (Table 1). On the other hand, phenylalanine, leucine, and lysine correlated positively with volatile compounds, except for 2,4-dimethylthiopene (Table 1).

The TPC also had significant correlations with all of the AOC assays. Specifically, it had a significant positive correlation with the DPPH and FRAP assays and a significant negative correlation with the ORAC assay (Table 2).

A significant positive correlation was observed between several amino acids, including threonine, arginine, tyrosine, valine, methionine, phenylalanine, isoleucine, leucine, lysine, and cystine, and the antioxidant capacity of wild garlic, as measured by DPPH radical scavenging and FRAP (Table 2). On the other hand, glycine, aspartic acid, and glutamic acid were significantly correlated with the ORAC assay. Only histidine was not significantly correlated with the antioxidant capacity (Table 2). 

All identified volatile compounds except diallyl trisulfide had a significant negative correlation with the ORAC assay. Diallyl sulfide, dimethyl trisulfide, and methyl-2-propenyl trisulfide had significant positive correlations, while 2,4-dimethylthiopene had a significant negative correlation with both the DPPH radical scavenging and FRAP assays (Table 2). 1,2-dithiole, 3-vinyl-1,2-dithi-4-ene, 4H-1,2,3-trithiin, and methyl-1,2,3,4-tetrathiane showed positive correlations only with DPPH radical scavenging, while allyl (Z)-1-propenyl disulfide and (E)-1-propenyl propyl disulfide had exclusive negative correlations with FRAP (Table 2).

### 3.2. Phytochemical Biodiversity of Wild Garlic Populations

The plant organ and location, as the main effects, and their interaction were found to be significant across all assays regarding the antioxidant capacity and total phenolic content (Table 3). The bulb samples from Prodin dol, Vukomerec, Dragonoš, and Novi Dvori exhibited significantly higher TPC than the leaf samples from those same locations (Table 3). Among the bulb samples, the bulbs from Vukomerec exhibited significantly higher TPC than the bulbs from other locations, except for the bulbs from Novi Dvori, which were found to be comparable (Table 3). Among the leaf samples, the plants from Rude, Prodin dol, Dragonoš, and Novi Dvori had significantly lower TPC values when compared to the other investigated locations (Table 3). Significant differences in the total antioxidant capacity, measured by DPPH assay, were determined between the bulb and leaf samples from Rude, Prodin dol, Vukomerec, Dragonoš, and Novi Dvori; the bulbs exhibited a significantly higher antioxidant capacity compared to the leaves (Table 3). The remaining locations showed comparable antioxidant capacity between the bulbs and leaves when measured by DPPH (Table 3). Among the bulb samples, the bulbs from Rude, Prodin dol, and Dragonoš exhibited the highest DPPH scavenging activity, closely followed by the bulbs from Novi Dvori and Vukomerec (Table 3). The bulbs from the other locations were found to be significantly lower in DPPH scavenging activity (Table 3). On the other hand, in the leaf samples, no significant differences among the locations were observed since all locations were found to be mutually comparable in the DPPH antioxidant capacity (Table 3).

When the antioxidant capacity was measured by FRAP, significantly higher values were determined in the bulbs compared to the leaves in the samples from Rude, Prodin Dol, Vukomerec, Dragonoš, and Novi Dvori, while the opposite—higher FRAP values in the leaf samples—were found in the samples from Motovun, Velanov Brijeg, and Grobnik (Table 3). In addition, among the bulb samples, the highest antioxidant capacity measured by FRAP was observed in the bulbs originating from Prodin dol, closely followed by the bulbs from Dragonoš, and then from Rude, Vukomerec, and Novi Dvori, while the lowest antioxidant capacity was measured in the bulb samples from Motovun, Velanov Brijeg, and Grobnik (Table 3). On the other hand, no significant differences among the locations in the leaf samples were observed (Table 3).

When the antioxidant capacity was measured by ORAC, the leaf samples across all locations exhibited significantly higher antioxidant capacity when compared to the bulb samples (Table 3). The bulb antioxidant capacity measured by ORAC was found to be significantly higher in the samples from Prodin Dol when compared to those from Motovun, Velanov Brijeg, Novi Dvori, and Grobnik but comparable to the samples from Rude, Vukomerec, and Dragonoš (Table 3). In the case of the leaf samples, the samples from Vukomerec exhibited a significantly higher antioxidant capacity measured by ORAC compared to those from the other locations, except for the samples from Dragonoš, which were found to be comparable to the Vukomerec samples (Table 3).

**Figure 2:**
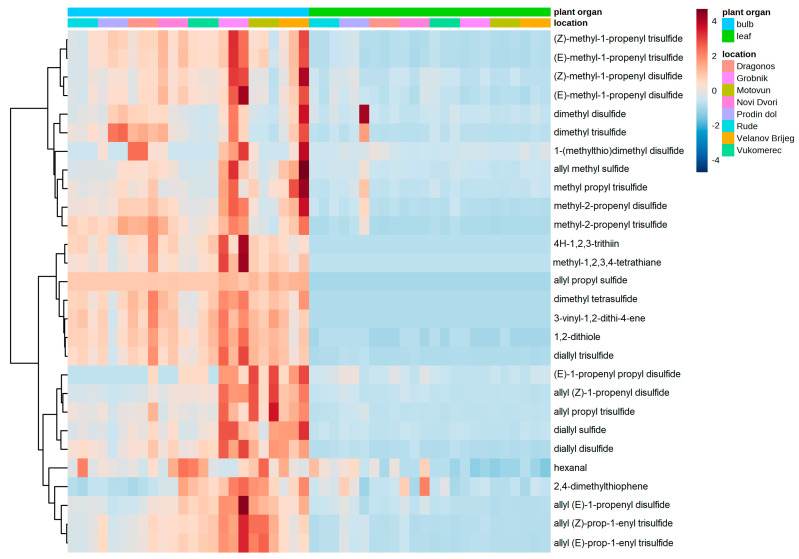
Volatile organic compounds identified in bulbs and leaves of wild garlic.

In total, 28 volatile organic compounds were identified and quantified, of which 27 were classified as organosulfur compounds and 1 was classified as an aldehyde (hexanal). As can be seen in Figure 1, the majority of volatile compounds were more abundant in the bulbs compared to the leaves. Furthermore, the bulb samples from Velanov Brijeg and Novi Dvori repeatedly stood out as samples exhibiting higher contents of the most volatile compounds compared to those from the other locations (Figure 2). The volatile compounds were clustered according to structural similarity into three groups. The first group included isomers of methyl-propenyl- (or methyl-allyl-) and dimethyl- disulifdes or trisulfides. An additional compound in this group was 1-(methylthio)dimethyl disulfide. The second group included compounds with sulfur atom cyclical structures, such as 4H-1,2,3-trithiin and methyl-1,2,3,4-tetrathiane, dimethyl tetrasulfide, allyl propyl sulfide, and diallyl trisulfide (Figure 2). The third group included mostly isomers of diallyl sulfide, disulfide, and trisulfide, with the addition of hexanal and 2,4-dimethylthiophene (Figure 2). The individual volatile compound contents and the analysis of variance among the plant organs and locations are presented in Supplementary Table S2.

**Figure 3:**
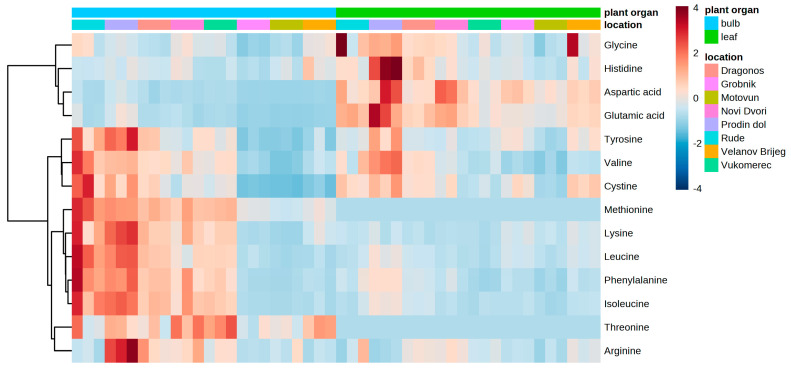
Amino acid contents in leaves and bulbs of wild garlic.

Out of the fourteen amino acids detected, five were classified as non-essential (aspartic acid, glutamic acid, glycine, arginine, and tyrosine), eight were classified as essential amino acids (histidine, threonine, valine, methionine, phenylalanine, isoleucine, leucine, and lysine), and cystine was the oxidized derivative of amino acid cysteine. According to Figure 3, the amino acid profiles were different between the bulbs and leaves of wild garlic, but also differences among the locations are evident (Figure 3). Glycine, histidine, aspartic acid, and glutamic acid were grouped together by the hierarchical clustering algorithm due to a higher content in the leaves compared to the bulbs from most of the investigated locations (Figure 3). These amino acids also exhibited higher biological variability in the leaves among locations compared to the bulbs (Figure 3). Tyrosine, valine, cystine, methionine, lysine, leucine, phenylalanine, isoleucine, threonine, and arginine were clustered together due to the higher bulb amino acid content, especially in Rude and Prodin dol (Figure 3).

The amino acid contents among the different plant organs and individual locations and the analysis of variance are presented in Supplementary Table S3. 

## 4. Discussion

The reallocation of phytochemicals between the plant organs is governed by their respective roles in cellular processes. The optimal defense theory, developed by McKey, states that the reallocation of secondary metabolites is focused on the defense of the most vulnerable or most important plant parts [26]. These metabolites should be inactive within the plant, but in the presence of an herbivore, these same metabolites should provide a significant degree of protection [27]. Regarding the determination of phytochemical parameters functioning as reallocation factors between the leaves and bulbs of wild garlic, among the investigated parameters, the VIP scores of methionine and threonine demonstrated high influence on the differentiation between the bulbs and leaves. Furthermore, a group of fifteen volatile compounds (allyl propyl sulfide, methyl-2-propenyl disulfide, (*E*)-methyl-1-propenyl disulfide, 1,2-dithiole, diallyl disulfide, methyl-2-propenyl trisulfide, (*Z*)-methyl-1-propenyl trisulfide, (*E*)-methyl-1-propenyl trisulfide, 3-vinyl-1,2-dithi-4-ene, 4H-1,2,3-trithiin, dimethyl tetrasulfide, allyl propyl trisulfide, allyl (*Z*)-prop-1-enyl trisulfide, allyl (*E*)-prop-1-enyl trisulfide, and methyl-1,2,3,4-tetrathiane) was found to be characteristic for the bulb samples due to having higher contents compared to the leaves. Due to the reallocation of resources from the leaves to the bulbs during bulb formation, a higher content of CSOs in bulbs, precursors for sulfur-containing volatile compounds, is expected [28]. On the other hand, higher ORAC, aspartic, and glutamic acid values were characteristic of the leaves of wild garlic, indicating the abundance of phytochemicals in the leaves of wild garlic.

In the last several decades, extensive research has been published on pathways for the biosynthesis of CSOs in *Allium* plants. First, radiotracer experiments have suggested that CSOs are biosynthesized from glutathione [29,30,31,32,33]. For the biosynthesis of CSOs (alliin, isoalliin, propiin, and methiin), the synthesis of an intermediate, namely glutathione from three amino acids, cysteine, glutamic acid, and glycine, is necessary [29,30,32,33]. According to Yoshimoto and Saito [31], the biosynthetic pathway for methiin is likely to be similar to those for alliin, isoalliin, and propiin. The generated intermediate is then converted into γ-glutamyl-S-1-propenylcysteine and/or γ-glutamyl-S-2-propenylcysteine, the precursors of isoalliin and alliin, respectively [31]. Basically, the formed CSOs are cleaved by alliinase to yield sulfenic acids, which spontaneously form allicin and many other thiosulfinates [3,6,31,34,35]. Thiosulfinates are also highly reactive and, as such, undergo further spontaneous reactions, giving rise to acyclic sulfides, dithiines, thiolanes, and ajoenes [4,31]. Our results regarding the correlation between amino acids and VOCs are in line with those of several authors who have proposed a pathway for the biosynthesis of CSOs, since our results show how the content of amino acids involved in the pathway is inversely proportional to the content of VOCs [4,7,18,36,37,38,39,40,41,42,43,44]. In addition to the glutathione precursor, volatile sulfur compounds can be synthetized from sulfur-containing amino acids, such as methionine. The positive correlation of methionine with many volatiles and the total phenolic content in our study, as well as its reallocation to the bulbs, indicates the importance of this compound in the metabolic pathways of wild garlic. Our investigation showed that the TPC correlated significantly with several amino acids, including phenylalanine, which is the precursor in the biosynthesis of many phenolic compounds in the shikimic acid metabolic pathway [37]. 

The antioxidant mechanism and antioxidant capacity of amino acids can vary due to structural differences in the side chains. Amino acids with aromatic rings/sulfur atoms/nitrogen atoms in their side chain are more vulnerable to an attack by an oxidant compared to other amino acids [38]. Our results show that several amino acids correlated positively with the TPC. The main pathway of phenolic compound synthesis is the shikimate pathway, in which phenylalanine is transformed via the phenylpropanoid and alkaloid biosynthesis pathways into numerous phenolic compounds and antioxidants, including those containing aromatic rings/sulfur atoms/nitrogen atoms in their side chain [37,39,40]. The principle of the TPC method is a reduction of the Folin–Ciocalteu reagent in the vicinity of a reducing agent, and amino acids containing aromatic rings/sulfur atoms/nitrogen atoms in their side chain can react with the Folin–Ciocalteu reagent, resulting in a color change, and hence, significant positive correlations between the TPC and phenylalanine, methionine, and lysine were observed [19,38]. These amino acids can be used by the cell as direct antioxidants. Since sulfur-containing amino acids show antioxidant properties, our results regarding the positive correlations among cystine, DPPH, and FRAP are in line with the results of Atmaca [44]. According to Xu et al. [38], tryptophan, methionine, histidine, lysine, cysteine, arginine, and tyrosine are considered antioxidative amino acids. Our results are partly in line with the mentioned study by Xu et al. [38], in the sense that only methionine and lysine were found to correlate with all three antioxidant capacity assays, while arginine, tyrosine, and cystine were found to correlate with the DPPH and FRAP assays. On the other hand, histidine was found to exhibit no significant correlation with the antioxidant capacity, although this amino acid is known to deliver considerable antioxidant properties [38]. The amino acids in plants have an important role in phytohormone biosynthesis, plant defense, and the production of secondary metabolites, all of which ensure the adaptation of the plant to its surrounding habitat [41,42,43].

The effects of interacting environmental factors can cause significant changes in the production and accumulation of secondary metabolites in plants, and these effects may be far from additive [45,46]. Our results show that in several locations, the TPC was higher in the bulbs compared to the leaves, but mostly, the phenolic content was comparable among the locations. The antioxidant capacity measured by DPPH radical scavenging and FRAP was higher in the bulbs compared to the leaves, while the ORAC assay had higher values in the leaves compared to the bulbs. In the studies published by Kovarovič et al. [11] and Lachowicz et al. [47] the leaf samples of wild garlic had higher TPC compared to the bulb samples. The differences in the wild garlic total polyphenol content can be attributed to the differences in physiological maturity, where higher values can be observed in late compared to early spring [9,47]. Other factors should be also taken into account in order to explain the content of polyphenols, such as the environmental conditions, the soil type and its characteristics, the nitrogen supply, and the surroundings of the collection site [48]. According to previously published studies by Mahmutović et al. [49], Mihaylova et al. [50], and Lachowicz et al. [47,51], wild garlic leaves exhibit significantly higher levels of antioxidant capacity compared to the bulbs. The availability of water is one of the known factors related to the variation in the production of metabolites in plants [45]. Since wild garlic prefers humid soils, water availability, in the form of either groundwater or rainfall, has most certainly affected the production of plant metabolites and, consequently, the antioxidant capacity [45,52]. According to Heimler et al. [48], the supply of nitrogen in the soil affects the phenolic content, and hence, the carbon/nitrogen balance pathways should be considered. Moreover, Jones et al. [53] reported that the nitrogen supply may influence sulfur uptake and the formation of CSOs, in the sense that the nitrogen metabolism is heavily intertwined with the sulfur metabolism. According to Vuković et al. [52], wild garlic also prefers soils well-supplied with nutrients. The nitrogen supply in the soil could have easily affected the contents of phenolic and organosulfur compounds in our samples, as well as the antioxidant capacity. 

The genus *Allium*, which includes wild garlic, has many species abundant in sulfur-containing volatile compounds, responsible for the characteristic aroma [1,54,55]. Therefore, similarities in the profiles of volatile organic compounds between garlic and wild garlic can be expected [4]. Many compounds detected in this study were also identified in garlic samples, which indicates the similarities between the volatile profiles of garlic and wild garlic [4]. In addition to the cysteine sulfoxide pattern and volatile profile, wild garlic also shares similarities with garlic in the amino acid profile [3,4,56]. According to several authors, the main amino acid in wild garlic is arginine, followed by aspartic acid, glutamic acid, methionine, threonine, valine, leucine, proline, and glycine [4,7,57]. Our study showed that the same amino acids were found to be abundant, although not in the order that other authors have reported. The observed differences among the amino acid contents could be due to the different origins of plants, the collection period, the environmental conditions, the soil type and its characteristics, the surroundings of the collection site, the organic matter in the soil (N supply), and the method applied [48,58,59,60,61].

Wild edible plants, wild garlic among them, often have an important contribution to the diet, bringing flavors that have been forgotten [12]. In particular, the use of wild edible plants in Europe has been especially related to periods of famine, when they were consumed as a life-saving food [12]. Consumers appreciate wild edible plants mainly because of their taste and aroma, with the added benefit of their great nutritional value, which is sometimes higher than several known common vegetables [12].

Moreover, numerous studies have shown that wild species are rich in secondary metabolites with great antioxidant and health-promoting properties, providing protection against a number of chronic and degenerative diseases [12,13,14,15,16,62].

## 5. Conclusions

The amino acids and total phenolic content were significantly correlated with the antioxidant capacity measured by DPPH radical scavenging and FRAP, indicating their important role in the antioxidant reaction in wild garlic. Several amino acids due to their structure greatly contribute to the antioxidant defense mechanism blurring the line between primary and secondary metabolites. Moreover, this study also confirms the significance of amino acids in the volatile organic compound biosynthesis pathway. All collected plants exhibited excellent bioactive properties regardless of the investigated locations. Each location represents its own biome with its altitude, surroundings, and local climatic conditions, and therefore, plants from different locations have different properties. Higher amino acid contents and antioxidant capacity were found in plants originating from Prodin dol, while higher volatile compound contents were found in plant material from Grobnik and Velanov Brijeg. In addition, higher total phenolic contents were found in plants originating from Vukomerec.

Based on the obtained results in this study and previous studies and the beneficial properties of wild garlic, there is reason to support the inclusion of this species in agronomic production. In addition to its beneficial properties, its wider use could greatly contribute to the biodiversity and sustainability of agriculture as well.

## Figures and Tables

**Table 1:** Pearson’s correlations among total phenolic content, volatile compounds, and amino acids in wild garlic.

Compound	Aspartic Acid	Glutamic Acid	Histidine	Glycine	Threonine	Arginine	Tyrosine	Valine	Methionine	Phenyl-Alanine	Isoleucine	Leucine	Lysine	Cystine
Total phenolic content	−0.27	−0.35	−0.25	−0.18	0.58	0.23	0.15	0.01	0.44	0.29	0.36	0.30	0.41	0.02
Allyl methyl sulfide	−0.41	−0.42	−0.09	−0.17	0.30	−0.06	−0.23	−0.11	0.15	−0.07	−0.07	−0.08	−0.01	−0.37
Hexanal	−0.36	−0.28	−0.02	0.05	0.42	0.09	−0.04	0.19	0.40	0.28	0.26	0.19	0.14	−0.01
Diallyl sulfide	−0.80	−0.77	−0.36	−0.40	0.74	0.29	0.06	0.08	0.73	0.42	0.47	0.41	0.46	−0.21
Allyl propyl sulfide	−0.54	−0.55	−0.20	−0.30	0.32	−0.11	−0.38	−0.23	0.17	−0.13	−0.13	−0.15	−0.08	−0.51
2,4-dimethylthiophene	−0.31	−0.28	−0.18	−0.25	0.26	−0.17	−0.45	−0.34	−0.07	−0.30	−0.27	−0.31	−0.29	−0.58
Dimethyl disulfide	−0.11	−0.19	0.26	0.01	0.22	0.08	0.14	0.22	0.16	0.12	0.12	0.11	0.13	−0.03
Methyl-2-propenyl disulfide	−0.52	−0.52	−0.13	−0.24	0.44	0.05	−0.09	0.02	0.36	0.12	0.13	0.11	0.17	−0.29
(Z)-methyl-1-propenyl disulfide	−0.47	−0.45	−0.21	−0.23	0.44	0.00	−0.20	−0.10	0.24	−0.01	0.02	−0.01	0.06	−0.39
(E)-methyl-1-propenyl disulfide	−0.49	−0.46	−0.21	−0.25	0.45	0.03	−0.16	−0.07	0.28	0.04	0.08	0.04	0.09	−0.37
1,2-dithiole	−0.72	−0.70	−0.33	−0.43	0.57	0.14	−0.09	0.03	0.60	0.30	0.32	0.27	0.30	−0.26
Diallyl disulfide	−0.66	−0.67	−0.28	−0.39	0.44	−0.04	−0.30	−0.15	0.37	0.05	0.06	0.02	0.05	−0.44
Allyl (Z)-1-propenyl disulfide	−0.54	−0.55	−0.24	−0.35	0.28	−0.13	−0.46	−0.34	0.09	−0.21	−0.19	−0.23	−0.19	−0.60
Allyl (E)-1-propenyl disulfide	−0.54	−0.52	−0.27	−0.35	0.37	−0.11	−0.38	−0.25	0.18	−0.11	−0.09	−0.13	−0.12	−0.52
(E)-1-propenyl propyl disulfide	−0.45	−0.43	−0.15	−0.25	0.24	−0.15	−0.45	−0.32	−0.01	−0.26	−0.25	−0.28	−0.24	−0.58
1-(methylthio)dimethyl disulfide	−0.39	−0.38	−0.16	−0.22	0.24	0.00	−0.17	−0.11	0.15	−0.05	−0.04	−0.03	0.00	−0.27
Dimethyl trisulfide	−0.36	−0.34	−0.01	−0.16	0.42	0.40	0.33	0.26	0.47	0.38	0.42	0.41	0.47	0.02
Methyl-2-propenyl trisulfide	−0.62	−0.59	−0.20	−0.31	0.59	0.27	0.15	0.20	0.64	0.42	0.44	0.41	0.46	−0.10
Methyl propyl trisulfide	−0.40	−0.43	−0.04	−0.17	0.34	−0.02	−0.13	−0.03	0.20	0.00	0.01	0.00	0.06	−0.32
(Z)-methyl-1-propenyl trisulfide	−0.61	−0.56	−0.26	−0.33	0.57	0.15	−0.08	−0.05	0.42	0.14	0.20	0.15	0.22	−0.36
(E)-methyl-1-propenyl trisulfide	−0.62	−0.58	−0.27	−0.34	0.57	0.13	−0.11	−0.08	0.40	0.11	0.17	0.12	0.19	−0.39
3-vinyl-1,2-dithi-4-ene	−0.74	−0.72	−0.34	−0.42	0.55	0.14	−0.11	0.01	0.60	0.30	0.31	0.27	0.29	−0.25
4H-1,2,3-trithiin	−0.64	−0.63	−0.34	−0.40	0.45	0.07	−0.14	−0.03	0.49	0.22	0.23	0.20	0.21	−0.26
Dimethyl tetrasulfide	−0.62	−0.61	−0.32	−0.41	0.37	−0.01	−0.25	−0.13	0.36	0.08	0.09	0.06	0.08	−0.38
Diallyl trisulfide	0.47	0.49	0.74	0.21	−0.24	−0.21	0.12	0.38	−0.22	0.01	−0.08	−0.10	−0.22	0.16
Allyl propyl trisulfide	−0.54	−0.56	−0.21	−0.34	0.26	−0.03	−0.33	−0.22	0.19	−0.07	−0.06	−0.09	−0.06	−0.48
Allyl (Z)-prop-1-enyl trisulfide	−0.60	−0.59	−0.25	−0.41	0.40	−0.03	−0.31	−0.23	0.26	−0.03	0.01	−0.06	−0.03	−0.49
Allyl (E)-prop-1-enyl trisulfide	−0.60	−0.59	−0.25	−0.40	0.41	−0.02	−0.29	−0.22	0.26	−0.02	0.02	−0.05	−0.02	−0.48
Methyl-1,2,3,4-tetrathiane	−0.74	−0.71	−0.34	−0.41	0.56	0.16	−0.10	0.00	0.58	0.28	0.30	0.26	0.30	−0.27

Significant correlations (*p* ≤ 0.05) are marked red.

**Table 2:** Pearson’s correlations between antioxidant capacity and bioactive compounds in wild garlic.

Compounds	DPPH	FRAP	ORAC
Total phenolic content	0.37	0.47	−0.30
Amino acids			
Aspartic acid	−0.37	−0.23	0.68
Glutamic acid	−0.28	−0.15	0.67
Histidine	−0.20	−0.13	0.26
Glycine	−0.11	−0.05	0.34
Threonine	0.46	0.47	−0.66
Arginine	0.55	0.69	−0.12
Tyrosine	0.66	0.72	0.05
Valine	0.59	0.56	−0.01
Methionine	0.88	0.75	−0.59
Phenylalanine	0.86	0.78	−0.30
Isoleucine	0.87	0.84	−0.34
Leucine	0.89	0.82	−0.28
Lysine	0.84	0.84	−0.32
Cystine	0.57	0.56	0.27
Volatile organic compounds			
Allyl methyl sulfide	−0.01	−0.15	−0.55
Hexanal	0.24	0.16	−0.58
Diallyl sulfide	0.48	0.32	−0.93
Allyl propyl sulfide	−0.09	−0.25	−0.69
2,4-dimethylthiophene	−0.31	−0.40	−0.52
Dimethyl disulfide	0.13	0.04	−0.32
Methyl-2-propenyl disulfide	0.20	0.02	−0.68
(Z)-methyl-1-propenyl disulfide	0.07	−0.08	−0.60
(E)-methyl-1-propenyl disulfide	0.12	−0.04	−0.62
1,2-dithiole	0.38	0.17	−0.88
Diallyl disulfide	0.10	−0.12	−0.83
Allyl (Z)-1-propenyl disulfide	−0.19	−0.38	−0.73
Allyl (E)-1-propenyl disulfide	−0.08	−0.27	−0.72
(E)-1-propenyl propyl disulfide	−0.28	−0.41	−0.63
1-(methylthio)dimethyl disulfide	0.10	−0.07	−0.45
Dimethyl trisulfide	0.47	0.37	−0.48
Methyl-2-propenyl trisulfide	0.53	0.35	−0.74
Methyl propyl trisulfide	0.01	−0.09	−0.56
(Z)-methyl-1-propenyl trisulfide	0.24	0.09	−0.75
(E)-methyl-1-propenyl trisulfide	0.20	0.06	−0.77
3-vinyl-1,2-dithi-4-ene	0.39	0.18	−0.87
4H-1,2,3-trithiin	0.29	0.08	−0.76
Dimethyl tetrasulfide	0.15	−0.06	−0.74
Diallyl trisulfide	−0.19	−0.21	0.05
Allyl propyl trisulfide	−0.06	−0.22	−0.71
Allyl (Z)-prop-1-enyl trisulfide	0.00	−0.18	−0.80
Allyl (E)-prop-1-enyl trisulfide	0.01	−0.17	−0.80
Methyl-1,2,3,4-tetrathiane	0.39	0.17	−0.86

Significant correlations (*p* ≤ 0.05) are marked red.

**Table 3:** Total phenolic content and antioxidant capacity of wild garlic bulbs and leaves from 8 separate locations (mean ± SE, *n =* 3).

Plant Organ	TPC mg GAE/g DW	DPPH	FRAP	ORAC nmol TE/g DW
		µmol TE/g DW		
Bulb	11.2 ± 0.3	9.80 ± 1.10	11.8 ± 1.0	87.2 ± 8.5
Leaf	10.4 ± 0.2	5.00 ± 0.20	8.80 ± 0.10	293 ± 10
*p*-value	***	***	***	***
Location				
Rude	10.1 ± 0.4 c ^1^	10.8 ± 2.4 a	11.3 ± 1.3 c	212 ± 43 ab
Prodin dol	10.7 ± 0.3 bc	10.0 ± 2.3 a	13.9 ± 2.2 a	195 ± 25 a–c
Vukomerec	12.0 ± 0.7 a	6.80 ± 1.10 b	11.5 ± 1.3 bc	229 ± 58 a
Motovun	11.0 ± 0.3 bc	4.00 ± 0.70 c	6.90 ± 0.90 e	165 ± 62 cd
Velanov Brijeg	10.7 ± 0.3 bc	4.30 ± 0.40 c	8.80 ± 0.60 d	165 ± 46 cd
Dragonoš	10.2 ± 0.5 c	10.0 ± 2.0 a	12.5 ± 1.7 b	222 ± 45 a
Novi Dvori	11.5 ± 0.4 ab	7.80 ± 1.70 b	10.6 ± 1.2 c	178 ± 47 b–d
Grobnik	10.3 ± 0.5 c	5.20 ± 0.30 c	6.80 ± 0.80 e	152 ± 51 d
*p*-value	**	***	***	***
Location	Bulb			
Rude	10.7 ± 0.2 c–f	15.9 ± 1.1 a	14.0 ± 0.8 c	117 ± 4 e–g
Prodin dol	11.4 ± 0.3 bc	15.0 ± 1.1 a	18.8 ± 0.2 a	146 ± 13 e
Vukomerec	13.2 ± 0.6 a	9.28 ± 0.63 c	14.2 ± 0.8 c	100 ± 8 e–g
Motovun	10.7 ± 0.3 c–f	3.15 ± 1.19 e	4.91 ± 0.35 f	26.1 ± 2.7 h
Velanov Brijeg	10.4 ± 0.5 c–g	3.90 ± 0.74 de	7.78 ± 0.87 e	66.2 ± 20.8 gh
Dragonoš	10.9 ± 0.2 c–e	14.4 ± 0.5 a	16.2 ± 0.8 b	120 ± 5 ef
Novi Dvori	12.4 ± 0.0 ab	11.4 ± 0.4 b	13.0 ± 1.0 c	72.9 ± 3.9 f–h
Grobnik	9.85 ± 0.80 e–g	4.85 ± 0.62 de	5.17 ± 0.36 f	46.3 ± 3.5 h
	Leaf			
Rude	9.48 ± 0.68 fg	5.61 ± 0.38 d	8.61 ± 0.32 de	306 ± 20 bc
Prodin dol	10.0 ± 0.0 d–g	4.97 ± 0.15 de	8.92 ± 0.13 de	245 ± 24 d
Vukomerec	10.6 ± 0.2 c–f	4.35 ± 0.19 de	8.67 ± 0.02 de	358 ± 17 a
Motovun	11.2 ± 0.6 b–d	4.92 ± 0.36 de	8.83 ± 0.30 de	304 ± 5 bc
Velanov Brijeg	10.9 ± 0.1 c–e	4.76 ± 0.41 de	9.76 ± 0.32 d	264 ± 25 cd
Dragonoš	9.36 ± 0.60 g	5.49 ± 0.22 d	8.74 ± 0.12 de	323 ± 5 ab
Novi Dvori	10.5 ± 0.1 c–g	4.18 ± 0.97 de	8.21 ± 0.24 de	283 ± 9 b–d
Grobnik	10.7 ± 0.5 c–f	5.61 ± 0.16 d	8.45 ± 0.18 de	257 ± 47 cd
*p*-value	**	***	***	**

^1^ Different letters indicate significant differences among groups after Fischer’s least significant difference post-hoc test; **—*p* ≤ 0.01; ***—*p* ≤ 0.001.

## Data Availability

The data are available within this article.

## Supplementary Materials

The following supporting information can be downloaded at: https://www.mdpi.com/article/10.3390/foods12112110/s1, Table S1: Location, coordinates, above sea level, and surroundings of the gathered plant material, Table S2: Volatile organic compounds identified in wild garlic bulbs and leaves from 8 different locations (mean ± SE, *n* = 3), Table S3: Amino acid content of wild garlic bulbs and leaves from 8 different locations (mean ± SE, *n* = 3).
